# Nicorandil protects mesenchymal stem cells against hypoxia and serum deprivation-induced apoptosis

**DOI:** 10.3892/ijmm.2015.2229

**Published:** 2015-05-29

**Authors:** FENGYUN ZHANG, JINJIN CUI, BO LV, BO YU

**Affiliations:** 1 Key Laboratories of Education Ministry for Myocardial Ischemia Mechanism and Treatment, The Second Affiliated Hospital of Harbin Medical University, Harbin 150086, P.R. China; 2 Department of Cardiology, The Second Affiliated Hospital of Harbin Medical University, Harbin 150086, P.R. China

**Keywords:** nicorandil, hypoxia and serum deprivation, apoptosis, phosphoinositide 3-kinase/Akt signaling pathway, reactive oxygen species, mesenchymal stem cells

## Abstract

Nicorandil, an adenosine triphosphate (ATP)-sensitive potassium (K_ATP_) channel opener, has been shown to exert a significant protective effect against ischemic heart injury. In the present study, we investigated the anti-apoptotic effects of nicorandil on rat mesenchymal stem cells (MSCs) subjected to hypoxia and serum deprivation (H/SD), as well as the potential underlying mechanisms. Apoptosis was induced in the MSCs by exposure to H/SD, and the apoptotic rates and reactive oxygen species (ROS) levels were determined by flow cytometry. The mitochondrial inner membrane potential was measured using the membrane-permeable dye, JC-1. Western blot analysis was used to measure the levels of Akt, Bcl-2, Bax, cytochrome *c* and cleaved caspase-3. The cell proliferative ability was assessed using the cell counting kit-8 (CCK-8) and 5-ethynyl-2′-deoxyuridine (EdU) assay. The results revealed that H/SD-induced apoptosis was significantly attenuated by treatment with nicorandil in a concentration-dependent manner. Moreover, nicorandil markedly reduced the levels of ROS which were induced by exposure to H/SD, and increased the stability of mitochondrial membrane potential and the Bcl-2/Bax ratio, while it concomitantly decreased the H/SD-induced cleavage of caspase-3 and the release of cytochrome *c*. Treatment with the phosphoinositide 3-kinase (PI3K) inhibitor, LY294002, abolished the beneficial effects of nicorandil on the MSCs. In conclusion, the findings of the present study indicate that nicorandil exerts protective effects against MSC apoptosis induced by H/SD and that these effects are mediated through the PI3K/Akt, mitochondrial and ROS signaling pathways.

## Introduction

Ischemic heart disease (IHD) is the leading cause of mortality worldwide, particularly in developed countries (1). Mesenchymal stem cells (MSCs) have been widely applied in regenerative medicine, with significant beneficial effects on post-infarction heart failure (2,3). However, the therapeutic potential of MSCs is impeded by their poor survival rate following transplantation into the harsh microenvironment of the infarcted myocardium. As shown in previous studies, the survival rates of human MSCs were <0.44% on day 4 following transplantation in immunodeficient mice (4) and <1% following autologous cell transplantation in humans (5). Thus, in order to improve the MSC-mediated beneficial effects on post-infarction heart failure, research should focus on finding strategies to increase the survival ability of donor MSCs. *In vivo*, the loss of nutritional factors and limited blood supply to the ischemic region are thought to be the key factors responsible for the high rate of MSC attrition (6). Correspondingly, hypoxia and serum deprivation (H/SD) have been designed to mimic the hostile ischemic microenvironment *in vitro* (6). Therefore, strategies that enhance survival under conditions of H/SD are pivotal for improving the efficacy of MSCs.

Adenosine triphosphate (ATP)-sensitive potassium (K_ATP_) channels are unique proteins that directly provide a link associating cellular energetics with electrical activity (7). K_ATP_ channels include the plasma membrane (sK_ATP_) and the inner mitochondrial membrane (mitoK_ATP_). Previous studies have suggested that mitoK_ATP_, rather than sK_ATP_ channels, are possible effectors of cardioprotection against ischemic injury (8,9). In addition, the mitoK_ATP_ channel has been shown to exert profound beneficial effects, including lowering of blood pressure, rectifying hypoglycemia, mimicking ischemic preconditioning, modifying arrhythmia like J-wave syndrome, atrial fibrillation and heart failure (10–12).

Nicorandil, a drug with both nitrate-like and K_ATP_ channel-activating properties, is the only mitoK_ATP_ channel opener used in clinical practice for the treatment of ischemic heart disease (13). Numerous experimental and clinical studies have reported the protective effects of nicorandil against myocardial ischemia (14,15), mainly in terms of limiting the size of the infarct area (16), reducing the no-reflow phenomenon (17), having anti-atherogenic properties (18) and inhibiting inflammation (19). Recently, nicorandil was reported to reduce the activation of the inflammasome and the subsequent release of caspase-1, interleukin (IL)-1β and IL-18 (20). However, the pro-survival effects of nicorandil on MSCs for regeneration purposes have not been examined thus far. In the present study, we hypothesized that nicorandil would prevent the apoptosis induced by exposure to H/SD and would thus improve the survival of MSCs. To confirm this theory, we examined the effects of nicorandil on the H/SD-induced apoptosis of MSCs and the related signaling pathways.

## Materials and methods

### Culture of MSCs

MSCs were isolated from the bone marrow of Sprague-Dawley (SD) rats (weighing 60–80 g) using a previously published method with minor modifications (21,22). All the SD rats were obtained from the Laboratory Animal Science Department, the Second Affiliated Hospital of Harbin Medical University, Harbin, China. The axperimental animal procedures were approved by the Local Ethics Committee for the Care and Use of Laboratory antimals of Harbin Medical University. Briefly, the femurs and tibias were removed from the SD rats, and the bone marrow was flushed out using 10 ml Iscove’s Modified Dulbecco’s medium (IMEM; Gibco, Grand Island, NY, USA) with 1% penicillin/streptomycin (Beyotime Institute of Biotechnology, Nantong, China). The cells were centrifuged at 300 x g for 5 min. The resulting cell pellets were resuspended in 6 ml IMEM supplemented with 10% fetal bovine serum (Gibco) and 1% penicillin/streptomycin and plated in a 25 cm^2^ plastic flask at 37°C in a humidified atmosphere containing 5% CO_2_ to allow the adherence of the MSCs. After 3 days, the medium was changed, and the non-adherent cells were removed. The medium was replaced every 3 days. Approximately 8–10 days after seeding, the cells became 80–90% confluent. The adherent cells were released from the dishes using 0.25% trypsin (Beyotime Institute of Biotechnology) and expanded at a 1:2 or 1:3 dilution. All subsequent experiments were performed using MSCs of passages 3–5.

For the identification of the MSC phenotype, the cells were harvested, washed with phosphate-buffered saline (PBS) and labeled with the following conjugated antibodies: FITC-labeled anti-CD44 (BD Pharmingen-550974; BD Biosciences, Franklin Lakes, NJ, USA), anti-CD45 (eBioscience-11-0461; eBioscience, San Diego, CA, USA), anti-CD29 (BD Pharmingen-555005; BD Biosciences), anti-CD34 (sc-7324; Santa Cruz Biotechnology, Inc., Dallas, TX, USA) and phycoerythrin-labeled anti-CD90 (BD Pharmingen-551401; BD Biosciences). The labeled cells were analyzed by flow cytometry and FACSDiva Pro Software (Becton-Dickinson, San Jose, CA, USA).

### Treatment of MSCs

The induction of apoptosis *in vitro* by H/SD, designed to mimic the *in vivo* conditions of the ischemic myocardium, was initiated as previously described by Zhu *et al* (6). In terms of the experimental design, the MSCs were washed with PBS, exposed to various concentrations (0, 10, 100, 500 and 1,000 *μ*M) of nicorandil (Sigma, St. Louis, MO, USA) in serum-free medium and incubated in a glove box (855-AC; Plas-Labs Inc., Lansing, MI, USA) to scavenge free oxygen at 37°C. Cells cultured in complete medium alone were used as the non-ischemic controls.

To investigate the mechanism of MSC apoptosis further, the phosphoinositide 3-kinase (PI3K) inhibitor, LY294002 (25 *μ*M; Cell Signaling Technology, Danvers, MA, USA), or the reactive oxygen species (ROS) scavenger, N-acetyl-L-cysteine (NAC) (500 *μ*M; Sigma-Aldrich, St. Louis, MO, USA), were added 1 h prior to treatment with nicorandil.

### Measurement of apoptosis and ROS levels

Cell death was assessed using the Annexin V-FITC/propidium iodide (PI) Apoptosis Detection kit (BD Biosciences). In accordance with the instructions provided by the manufacturer, the cells were harvested, washed with binding buffer (BD Biosciences) and resuspended in 200 *μ*l binding buffer. Annexin V solution (5 *μ*l; BD Biosciences) was added to the cell suspension followed by incubation for 20 min in the dark at 4°C. Subsequently, 5 *μ*l PI were added for 5 min, and the cell suspension was immediately analyzed by bivariate flow cytometry using BD FACSCanto II equipped with BD FACSDiva Software (Becton-Dickinson). Approximately 1×10^5^ cells were analyzed per sample. Annexin V^−^/PI^−^ staining represented surviving cells, Annexin V^+^/PI^−^ cells signified early apoptosis and PI^+^ cells indicated necrotic or apoptotic cells at the terminal stage.

Cellular ROS levels were determined using a Reactive Oxygen Species assay kit (Beyotime Institute of Biotechnology). Briefly, the cells were incubated with the diluted fluoroprobe, 2′,7′-dichlorodihydrofluorescein diacetate (DCFH-DA; Beyotime Institute of Biotechnology), for 20 min at 37°C with slight shaking every 5 min. After washing with serum-free culture medium, the cells were collected and examined by flow cytometry (FACSCanto II) at excitation and emission wavelengths of 488 and 525 nm, respectively, or examined under a fluorescence microscope (DMI4000B; Leica, Wetzlar, Germany).

### Detection of mitochondrial membrane potential (MMP or ΔΨm)

The loss of ΔΨm was determined using the JC-1 Mitochondrial Membrane Potential assay kit (Beyotime Institute of Biotechnology). Briefly, the MSCs were seeded in 6-well plates. Following treatment, the cells were washed with PBS and 5 *μ*M JC-1 was added, followed by incubation at 37°C for 20 min. Subsequently, the cells were washed twice with cold JC-1 staining buffer and visualized under a fluorescence microscope.

### Western blot analysis

At the end of the treatment period, the MSCs were harvested and lysed with ice-cold RIPA lysis buffer, and the homogenate was centrifuged at 12,000 × g for 10 min at 4°C. Total protein in the supernatant was quantified using a BCA Protein assay kit, and an aliquot (30–50 *μ*g) from each sample was separated by 12% sodium dodecyl sulfate-polyacrylamide gel electrophoresis (SDS-PAGE). The protein band was transferred onto polyvinylidene difluoride (PVDF) membranes blocked with 8% fat-free milk in Tris-buffered saline (TBS) with 0.5% Tween-20 for 60 min at 37°C, followed by treatment with the following primary antibodies at 4°C overnight: rabbit monoclonal against Akt (cst-4691s), phosphorylated Akt [p-Akt (Ser473); cst-4060s], caspase-3 (cst-9662s), Bax (cst-2772s), Bcl-2 (cst-2876s) and cytochrome *c* (cst-4272s) (all from Cell Signaling Technology) and mouse polyclonal antibody against β-actin (TA-09; Zhongshan Golden Bridge Biotechnology, Beijing, China). After washing in TBS with Tween-20 (TBS-T) buffer, the membranes were further incubated with horseradish peroxidase-conjugated anti-mouse (ZB-2305; Zhongshan Goldenbridge Biotechnology) and anti-rabbit (sc-2357) secondary antibodies (Santa Cruz Biotechnology, Inc.) for 60 min at 37°C. Subsequently, the membranes were washed in TBS-T solution 3 times, followed by the addition of TBS solution, and visualization using the ECL chemiluminescence detection system with BeyoECL Plus (Beyotime Institute of Biotechnology). Densitometric analysis of the protein bands was carried out using Quantity One software (Bio-Rad, Hercules, CA, USA).

### Cell viability and proliferation assay

Cell viability was assessed using the cell counting kit-8 (CCK-8). Briefly, the MSCs were cultured in 96-well plates at a density of 1,000 cells/well. Following an ~70% fusion of the cells, the indicated treatments were carried out. Subsequently, 10 *μ*l CCK-8 solution were added to each well and the plates incubated for 2 h. The absorbance at 450 nm was measured using a microplate reader (Tecan Infinite M200 microplate reader; LabX, Midland, ON, Canada). The mean optical density (OD) of 4 wells in each group was used to calculate the percentage of cell viability. The experiments were carried out in triplicate.

In order to determine the cell proliferative ability, the MSCs were cultured in 96-well plates for 24 h, followed by exposure to various concentrations of nicorandil for the indicated periods of time. An aliquot of 5-ethynyl-2′-deoxyuridine (EdU; 50 *μ*M; Ribobio, Guangzhou, China) was added to the culture medium for 2 h. The cells were washed with PBS, fixed with 4% paraformaldehyde, and dyed with Apollo staining reaction liquid (Ribobio). Hoechst 33342 (Ribobio) was used for the labeling of the nuclei. Images were acquired using a fluorescence microscope. Five fields were randomly selected from each dish, and at least 3 dishes were counted per concentration.

### Statistical analysis

Data were analyzed using SPSS 19.0 software (SPSS, Inc., Chicago, IL, USA). All values are expressed as the means ± SD. Differences among groups were examined by one-way ANOVA. The Student’s t-test was used to compare differences between 2 groups. Differences were considered statistically significant at P<0.05.

## Results

### Characterization of the rat MSCs

**Figure 1:**
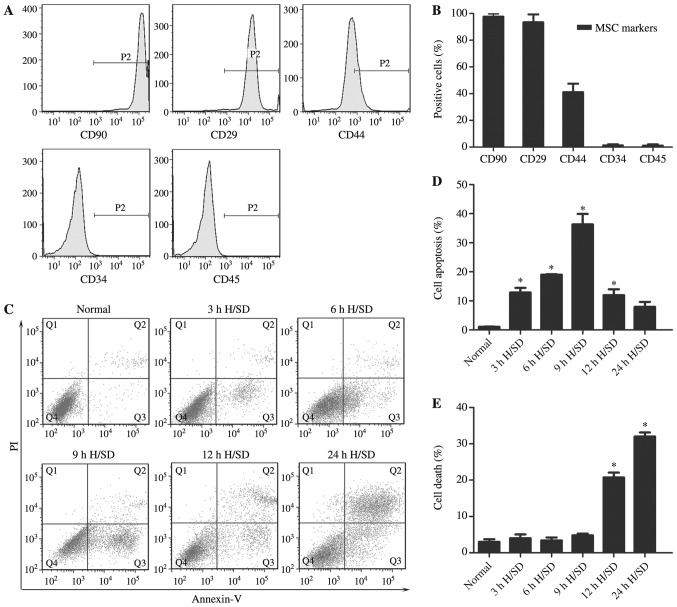
Apoptosis is induced in MSCs under H/SD conditions. (A and B) Cell surface makers of MSCs. (C–E) Analysis of apoptosis using flow cytometry for (D) early apoptotic cells and (E) necrotic or apoptotic cells in the terminal stages induced by H/SD in MSCs. Data are presented as the means ± SD of 3 separate experiments. ^*^P<0.05, compared with the normal group. MSCs, mesenchymal stem cells; H/SD, hypoxia/serum deprivation.

The MSCs obtained from the bone marrow of the SD rats exhibited a fibroblast-like appearance (data not shown). The results of flow cytometry revealed that the majority of the adherent MSCs from passage 3 expressed the common markers, CD90 (99.70±2.01%), CD29 (93.46±5.89%) and CD44 (41.16±6.27%), but were negative for CD34 (1.32±0.82%) and CD45 (1.15±0.88%) (Fig. 1A and B). Therefore, cells at passages 3–5 were used for the subsequent experiments.

### H/SD conditions induce the apoptosis of MSCs

The apoptosis of MSCs induced by H/SD (3–24 h) was examined (Fig. 1C). The early apoptotic rate was observed with a peak at 9 h in the MSCs exposed to H/SD (cells exposed to H/SD, 39.20±5.11% vs. normal cells, 1.07±0.11%; Fig. 1D). Following longer periods of treatment, the populations of PI^+^ cells representing necrotic or apoptotic cells at the terminal stage were significantly increased (Fig. 1E).

### Nicorandil protects MSCs against H/SD-induced apoptosis

**Figure 2:**
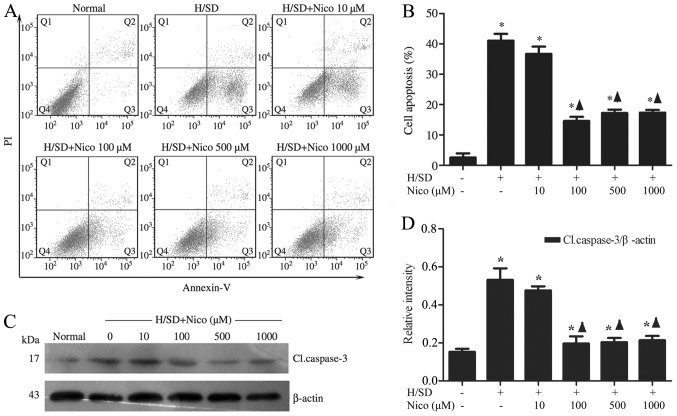
Nicorandil protects MSCs against H/SD-induced apoptosis. Cells were pre-incubated with nicorandil (10–1,000 *μ*M) for 1 h in complete medium prior to exposure to H/SD. Apoptosis was reduced by nicorandil in a dose-dependent manner, as assessed using (A and B) flow cytometry and (C and D) western blot analysis. Data are presented as the means ± SD of 3 separate experiments. ^*^P<0.05, compared with the normal group; ^▲^P<0.05, compared with the H/SD control group. MSCs, mesenchymal stem cells; H/SD, hypoxia/serum deprivation; Nico, nicorandil; Cl.caspase-3, cleaved caspase-3.

To determine whether nicorandil blocks the apoptotic process induced by H/SD, the MSCs were pre-treated with increasing concentrations of the drug (10–1,000 *μ*M) for 1 h, followed by exposure to H/SD for 9 h. The Annexin V^+^/PI^−^ MSC population, identified by flow cytometry, was reduced in the nicorandil-treated groups, particularly in the group treated with 100 *μ*M nicorandil [treated cells, 14.60±1.37% vs. apoptotic control (untreated cells), 41.10±2.20%], compared with the control group (Fig. 2A and B). In order to further elucidate the mechanisms underlying the anti-apoptotic effects exerted by nicorandil, western blot analysis was used to measure the expression levels of caspase-3, a known key mediator of apoptosis. Nicorandil significantly suppressed the cleavage of caspase-3 under H/SD conditions in a concentration-dependent manner, with the highest inhibitory effect observed in the group treated with 100 *μ*M nicorandil (treated cells, 0.20±0.04 vs. apoptotic control, 0.53±0.06; Fig. 2C and D).

### Nicorandil activates the PI3K/Akt signaling pathway in MSCs under H/SD conditions

**Figure 3:**
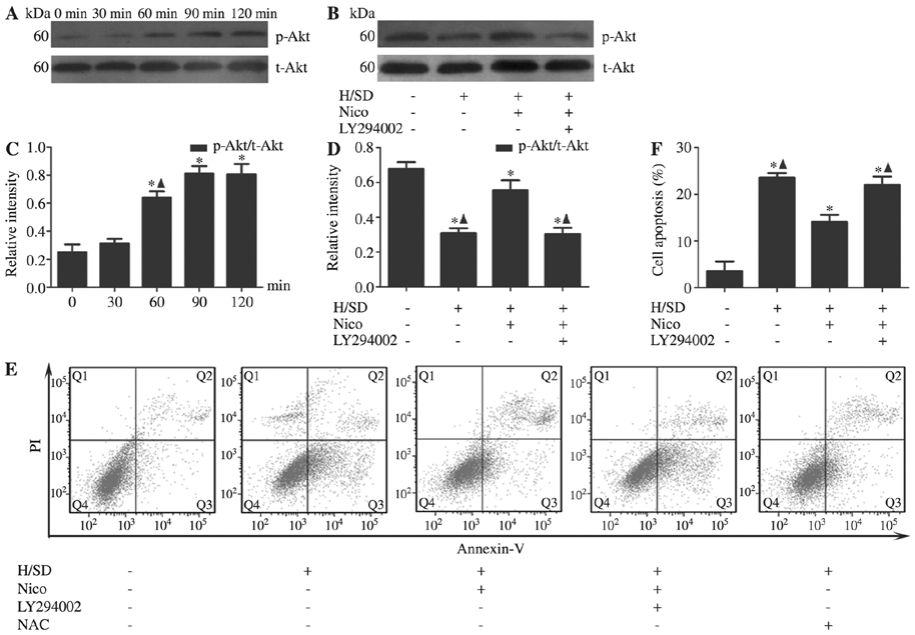
Nicorandil activates the PI3K/Akt signaling pathway under H/SD conditions. MSCs were treated with nicorandil (100 *μ*M) for the indicated periods of under H/SD conditions for 9 h. (A and C) The levels of p-Akt was upregulated by nicorandil in a time-dependent manner, peaking at 90 min. Data are presented as the means ± SD of 3 separate experiments. ^*^P<0.05, compared with the normal group; ^▲^P<0.05, compared with the group treated with nicorandil for 90 min. (B and D) The inhibition of PI3K with LY294002 triggered p-Akt inactivation. (E and F) Nicorandil induced a significant decrease in the apoptotic rate of MSCs under H/SD conditions, which was reversed by LY294002. Data are presented as the means ± SD of 3 separate experiments. ^*^P<0.05, compared with the normal group; ^▲^P<0.05, compared with the 100 *μ*M nicorandil-treated group. PI3K, phosphoinositide 3-kinase; MSCs, mesenchymal stem cells; H/SD, hypoxia/serum deprivation; Nico, nicorandil; p-Akt, phosphorylated Akt (Ser473); t-Akt, total Akt; NAC, N-acetyl-L-cysteine.

Following validation of the anti-apoptotic effect of nicorandil on MSCs under H/SD conditions, the underlying mechanisms were explored. In view of the finding that PI3K/Akt signaling protects the heart against ischemic injury, we examined the association between nicorandil and the PI3K/Akt pathway. The MSCs were treated with nicorandil (100 *μ*M) for the indicated periods of time (0, 30, 60, 90 and 120 min). Compared with the control group, we observed that the activation of Akt, as evident from the increased levels of p-Akt at Ser473 by nicorandil in a time-dependent manner, peaked at 90 min [Akt (Ser473) 90 min, 0.81±0.05 vs. 0 min, 0.25±0.60] (Fig. 3A and C). To further confirm the role of this pathway in the anti-apoptotic effects of nicorandil, PI3K/Akt was blocked using the PI3K-specific inhibitor, LY294002. The results from western blot analysis revealed that the inhibition of Akt expression by LY294002 [Akt (Ser473) with LY294002, 0.30±0.04 vs. without LY294002, 0.55±0.06] in the group treated with 100 *μ*M nicorandil (Fig. 3B and D). Moreover, co-incubation with LY294002 partially abrogated the anti-apoptotic effects of nicorandil on the MSCs, as evidenced by the increase in the number of Annexin V^+^/PI^−^ cells (with LY294002, 21.99±1.83% vs. without LY294002, 14.07±1.52%; Fig. 3E and F).

### Nicorandil exerts anti-apoptotic effects by stabilizing the MMP

**Figure 4:**
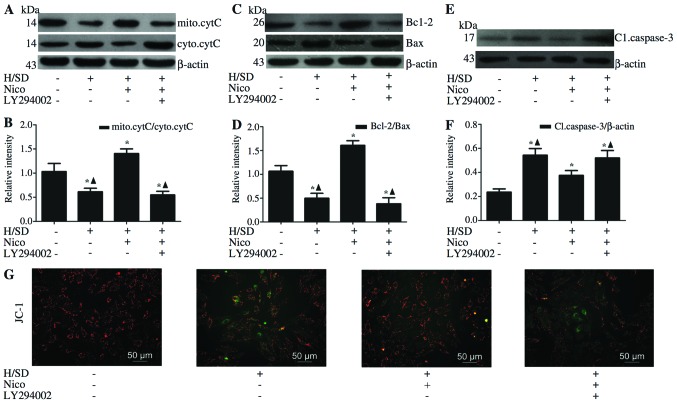
Nicorandil exerts anti-apoptotic effects by stabilizing MMP. Western blot analysis revealed that nicorandil induced a significant increase in (C and D) the expression of anti-apoptotic Bcl-2, with a concomitant decrease in (A–D) the pro-apoptotic proteins Bax and cytochrome *c*, as well as apoptosis-related (E and F) capsase-3, and these effects were reversed by LY294002 [an inhibitor of phosphoinositide 3-kinase (PI3K)]. (G) Nicorandil exerted a significant inhibitory effect on mitochondrial dysfunction, as verified by JC-1 staining. Data are presented as the means ± SD of 3 separate experiments. ^*^P<0.05, compared with the normal group; ^▲^P<0.05, compared with the 100 *μ*M nicorandil-treated group. H/SD, hypoxia/serum deprivation; Nico, nicorandil; MMP, mitochondrial membrane potential; mito.cytC, mitochondrial cytochrome *c*; cyto.cytC, cytosolic cytochrome *c*; Cl.caspase-3, cleaved caspase-3.

Depolarization of the inner MMP is a sign of cell death (23). Therefore, in order to ascertain whether nicorandil preserves mitochondrial integrity through the maintenance of MMP, we performed JC-1 staining. As shown in Fig. 4G, the red/green ratio of JC-1 was decreased in the MSCs exposed to H/SD compared with the normal group, and this effect was reversed by nicorandil, particularly in the group treated with 100 *μ*M nicorandil, which is consistent with the Annexin V-PI measurements (Fig. 3E and F). These results confirm the beneficial effects of nicorandil on mitochondrial function. The integrity of the mitochondrial membrane affects the release of pro-apoptotic cytochrome *c* from the mitochondria to the cytosol (24). Western blot analysis revealed the inhibition of the release of cytochrome *c* in the nicorandil-treated group, compared with the group exposed to H/SD (levels of mitochondrial cytochrome *c*: treated cells, 1.41±0.10 vs. apoptotic control, 0.61±0.07; Fig. 4A and B). To further determine whether the anti-apoptotic effects of nicorandil involve the inhibition of the mitochondrial pathway in the MSCs exposed to H/SD, the Bcl-2/Bax ratio, which plays an important role in mitochondrial integrity (25), was examined by western blot analysis (Fig. 4C and D). Compared with the cells exposed to H/SD, the Bcl-2/Bax ratio was significantly increased in the group treated with 100 *μ*M nicorandil (treated cells, 1.61±0.10 vs. apoptotic control, 0.50±0.10; Fig. 4C and D), and the levels of caspase-3, a key mediator of apoptosis, were decreased (treated cells, 0.37±0.04 vs. apoptotic control, 0.54±0.06; Fig. 4E and F).

### Involvement of ROS in the nicorandil-mediated protective effects against the apoptosis of MSCs exposed to H/SD

**Figure 5:**
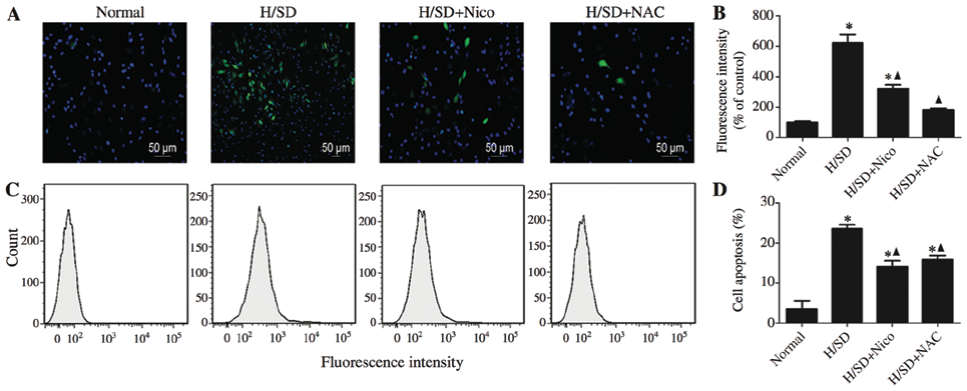
Involvement of ROS in the protective effects of nicorandil against the H/SD-induced apoptosis of MSCs. MSCs were pre-treated with nicorandil (100 *μ*M) or NAC (500 mM) for 1 h, followed by exposure to H/SD for 9 h. Intracellular ROS were visualized using (A) fluorescence microscopy and (B and C) flow cytometry. (D) Mean data values from the apoptotic cells are shown. The apoptotic rate was measured by flow cytometry following Annexin V and PI staining. Data are presented as the means ± SD of 3 separate experiments. ^*^P<0.05, compared with the normal group; ^▲^P<0.05, compared with the 100 *μ*M nicorandil-treated group. MSCs, mesenchymal stem cells; H/SD, hypoxia/serum deprivation; ROS, reactive oxygen species.

ROS function as pivotal components in pro-apoptotic signaling cascades (26). In this study, MSCs exposed to H/SD displayed an approximate 7-fold increase in ROS production, compared with the untreated cells (Fig. 5A and B). Nicorandil induced a significant inhibition of ROS production, as was determined by the DCFH oxidation assay. Similar results were obtained with the general ROS scavenger, NAC (500 *μ*M) (nicorandil-treated cells, 321.00±25.71 vs. apoptotic control, 621.98±55.29; NAC-treated cells, 180.33±11.72 vs. apoptotic control, 621.98±55.29; Fig. 5A and B). Consistently, the results of flow cytometry revealed that pre-treatment with either nicorandil or NAC for 1 h prior to exposure to H/SD, induced a significant decrease in apoptosis (nicorandil-treated cells, 14.07±1.51% vs. apoptotic control, 23.57±1.00%; NAC-treated cells, 15.87±0.95% vs. apoptotic control, 23.57±1.00%; Figs. 3E and 5C and D), indicating that nicorandil confers its protective effects partly by decreasing ROS production.

### Nicorandil has little effect on MSC proliferation

**Figure 6:**
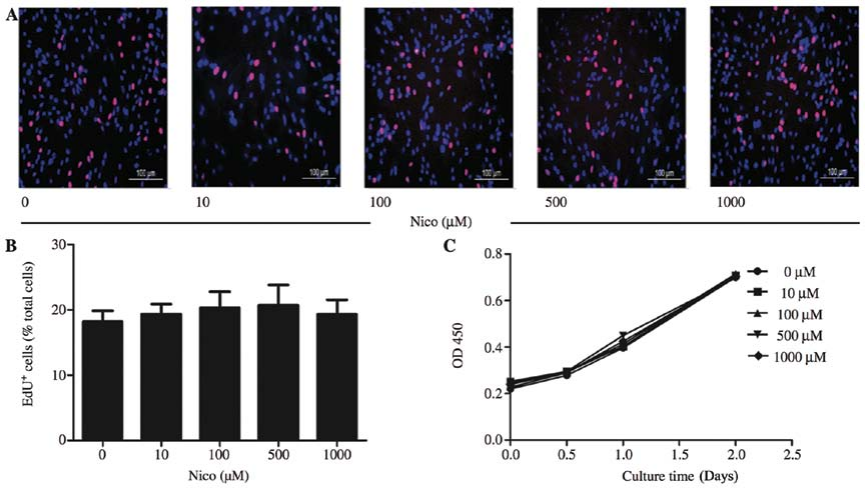
Nicorandil exerts little effect on MSC proliferation. Various concentrations of nicorandil were added to the culture medium for the indicated periods of time in order to examine its effects on MSC viability and proliferation. (A and B) The EdU assay indicated that nicorandil had little effect on MSC proliferation, even at >1,000 *μ*M, and did not induce cell toxicity (P>0.05). (C) Growth curves of MSCs obtained by CCK-8 assay, indicating no significant changes during the 2 days of treatment with various concentrations of nicorandil. Data are presented as the means ± SD of 3 separate experiments. Nico, nicorandil; MSCs, mesenchymal stem cells.

To the best of our knowledge, the effects of nicorandil on MSC proliferation have not been documented to date. Therefore, we examined the effects of nicorandil at the aforementioned range of concentrations on MSC viability using the CCK-8 assay and EdU staining. Data from EdU staining indicated that nicorandil had little effect on MSC proliferation (Fig. 6A and B). In accordance with the results of EdU staining, the proliferation growth curves of the MSCs obtained by CCK-8 assay did not show any significant changes during the 2 days of nicorandil treatment at any of the concentrations used (Fig. 6C).

## Discussion

In the present study, we demonstrated that nicorandil suppressed the apoptosis of MSCs exposed to H/SD in a dose-dependent manner. Moreover, the anti-apoptotic effects of nicorandil were shown to be mediated through the PI3K/Akt, mitochondrial and ROS signaling pathways. To the best of our knowledge, this is the first study to report the protective effects of nicorandil on MSCs under H/SD conditions *in vitro* and the underlying mechanisms.

Since MSCs are easily obtained and exhibit impressive paracrine ability, multilineage differentiation potential (27) and the capacity to activate endogenous cardiac progenitor cells (CPCs) (28), they represent one of the most ideal seed cell candidates for tissue regeneration in several clinical diseases, including myocardial infarction (3). However, the therapeutic potential of MSCs is limited by their poor survival rate following transplantation into the deleterious infarcted myocardium (29). Zhang *et al* (30) demonstrated that stem cell therapy only slightly improved cardiac function, and the majority of the implanted MSCs in the infarcted myocardium died within 7 days. Therefore, the high rate of attrition of transplanted MSCs is a primary concern. Studies have demonstrated that apoptosis is the main pattern of cell death when MSCs are exposed to H/SD *in vitro*, mimicking the *in vivo* microenvironment of ischemic injury (6,31).

Nicorandil is the only mitoK_ATP_ channel opener currently used in clinical practice as a cardioprotective drug. Experimental and clinical studies have described the protective effects of nicorandil against ischemic heart disease (14,15). Recently, other cytoprotective effects of nicorandil have been reported. Izumiya *et al* (18) demonstrated that the treatment of ApoE^−^ deficient mice on an atherogenic diet for 8 weeks with nicorandil significantly reduced atherosclerotic lesions and plaque necrosis. Moreover, nicorandil attenuated tunicamycin-induced C/EBP homologous protein (CHOP) expression in cultured THP-1 macrophages (18). Nicorandil has also been shown to reduce the activation of the inflammasome and, subsequently, the release of caspase-1 and the levels of IL-1β and IL-18 (20). The majority of these studies have demonstrated that the opening of mitoK_ATP_ channels is one of the most important mechanisms underlying the protective effects of nicorandil. Kim *et al* (15) reported that nicorandil inhibited apoptosis induced by oxidative stress through the activation of mitoK_ATP_ channels, partly by preserving ΔΨm. The mitoK_ATP_ channel opener, diazoxide, has recently been shown to improve the survival ability of MSCs *in vivo* and *in vitro* when these are transplanted into rats with acute myocardial infarction or are subjected to oxidative stress with 100 *μ*M H_2_O_2_ (preconditioned MSCs) by improving mitochondrial function (32,33). Based on these findings, we hypothesized that nicorandil may exert protective effects on MSCs under H/SD conditions by activating the mitoK_ATP_ channels.

In our experiments, the apoptosis of the MSCs exposed to H/SD significantly increased, compared to the normal group. Notably, pre-treatment with nicorandil at concentrations ranging from 10 to 1,000 *μ*M for 1 h and exposure to H/SD for 9 h led to decreased apoptosis in a concentration-dependent manner. Hoever, further studies are required to explore the molecular mechanisms underlying the regulation of apoptosis by nicorandil.

We further examined whether the PI3K/Akt signaling pathway is involved in the anti-apoptotic activity of nicorandil in MSCs. The PI3K/Akt pathway plays an essential role in promoting survival in various cell types (34,35). Our results demonstrated that nicorandil increased Akt phosphorylation and these protective effects were effectively blocked by a PI3K inhibitor (LY294002), strongly suggesting an essential role for the PI3K/Akt signaling pathway in the nicorandil-mediated protection of MSCs exposed to H/SD.

As an inevitable by-product of mitochondrial respiration, ROS are mainly produced in the mitochondria under normal conditions, and moderate amounts are necessary for cell survival, proliferation and pro-longevity (36). However, during hypoxia, an imbalance between the formation and scavenging of free radicals leads to the overproduction of electrons. These electrons react with remnant molecular oxygen, leading to ROS generation (37). Plethoric ROS may result in the loss of MMP, the release of pro-apoptotic molecules and the initiation of caspase-dependent apoptosis (26). Previous studies have demonstrated that the regulation of the mitochondrial potassium membrane permeability contributes to the mitoK_ATP_ opener-mediated suppression of ROS production (38). Our findings indicated that H/SD induced the production of excess ROS, and this effect was suppressed by treatment with nicorandil.

Our study had several limitations. Firstly, the H/SD model is an *in vitro* experimental representation of acute myocardial infarction, which fails to completely mimic the *in vivo* ischemic and inflammatory microenvironment. Further studies are warranted to ensure the comprehensive understanding of the cellular mechanisms involved, both *in vitro* and *in vivo*. Secondly, apoptosis is only one of the processes that damage MSCs during H/SD. Other factors involving inflammation may also lead to the deterioration of the survival ability of MSCs. Recently, nicorandil was reported to reduce the activation of the inflammasome and the release of caspase-1, IL-1β and IL-18, following oxygen-glucose deprivation-induced inflammation in BV-2 cells (20). Therefore, we need to focus on the role of inflammasomes in MSCs under H/SD conditions and on the related effects of nicorandil. Thirdly, multiple mechanisms and pathways, such as 5′ adenosine monophosphate-activated protein kinase (AMPK)/endothelial nitric oxide synthase (eNOS), participate in sustaining and mediating apoptosis (39), and thus require further investigation.

In conclusion, the results of the present study provide preliminary evidence indicating that nicorandil promotes MSC survival under conditions mimicking the myocardial ischemia. The pro-survival effects of nicorandil against H/SD-induced mitochondrial apoptosis are possibly a result of the activation of the PI3K/Akt signaling pathway and the reduction of ROS production.
